# Connecticut Valley Dental Association

**Published:** 1864-06

**Authors:** L. D. Shepard


### Proceedings of Societies.

### CONNECTICUT VALLEY DENTAL ASSOCIATION.

BY L. D. SHEPARD, D.D.S.

New England is just waking up to the advantages of associative effort in promoting the interests of dental science.

Some ten years since a Society was formed in New Hampshire, and also one in Vermont, which held a meeting or two and died, since which time, until within the past year, New England has had no active Society.

The past year has witnessed a decided revival. In it have been formed the Merrimac Valley, New Haven and Connecticut Valley Associations, and others are talked of.

The Connecticut Valley Association was organized at Springfield, Mass., Nov. 10, 1863. Its origin more properly dates from the meeting of the American Convention at Saratoga, in August, for here the subject was canvassed, and the move determined upon by a half dozen dentists representing Western New England, who felt the need of such an Association.

The first meeting was well attended. An unusual degree of unanimity, zeal and devotedness were manifested, and, in one evening, a Constitution and By-Laws were drawn up, discussed and adopted, and the organization completed by the election of the following officers for the ensuing year:

*President*—Dr. F. Searle, Springfield, Mass.

*Vice-Presidents*—Dr. O. R. Post, Brattleboro, Vt.; Dr. C. Stratton, Amherst, Mass.

*Secretary*—Dr. L. D. Shepard, Amherst, Mass.

*Treasurer*—Dr. H. M. Miller, Westfield, Mass.

*Executive Committee*—Dr. E. V. N. Harwood, Rutland, Vt.; Dr. C. S. Hurlbut, Springfield, Mass.; Dr. A. A. Howland, Barre, Mass.

The second meeting was held at Greenfield, Mass., on the 12th and 13th of January, 1864. Nearly all the old members were present, and seven new members were admitted.

The following topics were discussed with much interest:

Causes of Decay in Teeth.

Filling Teeth.

Anæsthetics.

The latter part of Tuesday evening was devoted to sociality. Our hearts were warmed towards one another by the magnetism of a fine supper. After the teeth had done their appropriate work, the tongue held sway for a couple of hours, and, at a late hour, we parted, feeling that we were no longer strangers, but brothers laboring in a common cause, in which the hopes, disappointments and victories of each were the property of all.

The third meeting was held at Hartford, Connecticut, on May 6th.

The New Haven Society, by invitation, met with us. Ten new members were admitted.

The regular order of exercises was varied somewhat, to listen to the valuable instructions of Profs. J. Taft, of Cincinnati, and W. H. Atkinson, of New York.

Dr. Taft referred to the rapid progress of the profession during the past few years, and showed the many advantages to be derived from associated effort. While the national organization is a source of profit to all, the main benefits grow out of the local associations, serving to strengthen the bond of fellowship between members, and enabling each and every one to gain in knowledge by the experience of others, thus continually elevating the standard of professional skill. He spoke also of the necessity of educating the people to an appreciation of their teeth, and the importance of good operations. His remarks were listened to with attention by all, and the favor with which they were received was manifested by frequent applause.

Dr. Atkinson agreed with Dr. Taft, as to the importance of Associations, and gave an interesting account of his own experience, showing that years of patient study are necessary to understand the true theory of the government of health.

The afternoon session was devoted to the discussion of Alveolar Abscess.

In the evening, Anæsthesia was the subject.

The following members were elected to represent the Connecticut Valley Association at the American Association in July:

Dr. F. Searle, Springfield, Mass.; Dr. L. D. Shepard,; Amherst, Mass.; Dr. Jonas McManus, Hartford, Conn. Dr. J. Beals, Greenfield, Mass.; Dr. E. V. N. Harwood, Rutland, Vt.; Dr. C. S. Hurlbut, Springfield, Mass.; Dr. W. H. Jones, Northampton, Mass.

The following vote was unanimously passed:

*Resolved*, That the members of the Connecticut Valley Dental Association tender their sincere thanks to Drs. Taft and Atkinson for their presence and highly interesting remarks during the sessions of this convention.

The next meeting will be held at Brattleboro, Vermont, on the second Tuesday in October.

After the adjournment, the members were handsomely entertained at the residence of James H. Ashmead, of Gold Foil fame.

